# Effect of gibberellic acid on the quality of sperm and *in vitro* fertilization outcome in adult male rats

**Published:** 2013

**Authors:** Mohammadreza Hosseinchi, Farhad Soltanalinejad, Gholamreza Najafi, Leila Roshangar

**Affiliations:** 1 *Department of Basic Sciences, Faculty of Veterinary Medicine, Urmia University, Urmia, Iran;*; 2 *Department of Anatomical Sciences, Faculty of Medicine, Tabriz University of Medical Sciences, Tabriz, Iran.*

**Keywords:** Chromatin, Fertility, Gibberellic acid, Rat, Sperm

## Abstract

Gibberellic acid (GA3) is a group of plant hormones identified in various plants. The aim of this study was to determine the effects of GA3 on sperm parameters and *in vitro* fertilization (IVF). Fifty six adult male rats were divided into seven groups as, control, treatment and sham. Following 15, 30 and 45 days of GA3 and methanol alcohol (MA) administration, rats were euthanized and epididymis tail was transferred to human tubular fluid (HTF) medium containing 4 mg mL^-1^ bovine serum albumin (BSA) .Total number of sperms, the percentage of live sperms, immature sperms and sperms with damaged chromatin and IVF were examined. The oocytes were obtained from immature rats after the injection of pregnant mare's serum (PMSG) and human chorionic gonadotropin (HCG) hormones. Human tubular fluid was used as the fertilization medium and zygotes transferred to fresh 1-cell rat embryos culture medium (mR1ECM) to reach the blastocyst stage. This study showed that GA3 could decrease the number of total sperms on days 30 and 45 in treated group comparison with the control and sham groups. Additionally, GA3 increased the immature sperms and sperms with damaged chromatin. The percentage of fertilization, two-cell embryos and blastocyst resulting from the treatment group on days 30 and 45 also decreased and showed significant differences with the control and sham groups (*p* < 0.05). The results obtained from this study indicated that the oral use of GA3 could reduce the fertility in rats by influencing the sperm number and the quality of sperm’s chromatins.

## Introduction

Gibberellic acid (a group of related substances called gibberellins) was discovered as a metabolic byproduct of the fungus *Gibberellafujikuroi*.^1^ Gibberellic acid has many effects regulating various physiological processes, including seed germination, the mobilization of endo-sperm storage reserves, shoot growth, flowering, floral development and fruit set.^2^^,^^3^ Moreover, El-Mofty *et al*. reported that GA3 is a plant growth regulators (PGRs) used in many countries, including Egypt to increase the growth of fruits and vegetables.^4^ This hormone is also used in Iran for increasing fruitage.

The feeding of GA3 to the house mouse (*Mus* *musculus*) doubled the proportion of females producing litters without increasing in litter size or number.^5^ Gibberellic acid had positive influence of on body weight, food conversion rate and fecundity on rat and mice.^6^^,^^7^ Gawienowski *et al*. demonstrated that GA3 exhibited synergistic uteropic effect with estradiol in the immature mouse.^8^ Treatment of neonatal rats with a single dose of gibberellin had a notable influence on certain endocrine indices in the adulthood. It accelerated the growth rate of the animals and produced weight increase of thyroid, ovary and adrenals as well as of the blood calcium level, but reduced the weight of the testis.^9^ GA3 stimulates the growth of the comb in the male chicks, but is ineffective in females. Estrogen prevents gibberellin-induced stimulation.^10^

The administration of GA_3 _decreases the activity of alanine aminotransferase and aspartate aminotransferase enzymes in the semen of the rabbits. Also, a gradual and significant decrease in serum testosterone concentration has been observed in male rabbits administered with GA_3._^11^ Gibberellic acid decreases the amount of total protein and alkaline phosphatase (ALP) and increases the amount of hexokinase and phosphatase acid in rats.^6^

Several studies demonstrated that in animals, chronic GA3 consumption increased tumor formation and oxidative stress.^12^ The lipid peroxidation end product i.e. malondialdehyde (MDA) significantly increased in the spleen and lungs of rats treated with abscisic acid (ABA) and GA_3_. Studies concluded that administration of subacute ABA and GA_3_ promoted lipid peroxidation content and altered the antioxidative systems in the rat's various tissues. Plant growth regulator produced oxidative stress in the spleen and lungs of rats during the period of a 25-day sub-chronic exposure.^12^^,^^13^ Significant increase in malondialdehyde and decrease in oxidant enzymes such as catalase (CAT) and superoxide dismutase (SOD), glutathione peroxidase (GPX) are confirming the hepatotoxicity induced by GA3 on rat liver. Troudi *et al*. showed that GA3 is hepatotoxic and causes histopathologic changes in the liver of rats and their neonates.^14^

Nassar *et al*. demonstrated that the sperm abnormalities in rats induced by GA3-treatment, involved the head or the tail region and/or head and tail together. Head abnormalities were presented in various forms. In some cases, the size of the head was within the normal limits, while in others the size was abnormal or it had an irregular outline which was rather difficult to describe. The sperm abnormalities include coiled tail, detached head and sperm without tail. Also the abnormalities may be occurring in both head and tail together.^15^ Rats that were given GA3 both orally and intradermally for 45 days showed decreased total protein content and ALP activity in their testicular cell preparation.

Histological studies showed loss of germ cells, derangement of the germinal cells, and reduction in the size of the seminiferous tubules and dystrophy of Leydig cells. More importantly decreased sperm count in the lumen was observed.^6^

Gibberllins, are claimed to be relatively harmless for animals and human. However, as mentioned, they have adverse effects on various animal tissues. People may be exposed to residues of GA3 in diet derived from consumption of different types of fruits and vegetables treated with GA3. Exposure to residues may also happen through drinking water.^16^ Occupational exposure of the agricultural workers to GA_3_ may occur through inhalation of powder and dermal contact with this compound at work places where GA_3 _is produced or used giving the picture of acute toxicity.^17^

Thus, the aim of this study was to evaluate effects of this hormone on sperm quality and fertility in rats.

## Materials and Methods

In this study, 56 Sprague Dawley male adult rats weighting 180 ± 10 g were used and divided into seven groups. Group 1 served as a control group which was not treated (n = 8). Groups 2, 3 and 4 were administered orally with 10 mg kg^-1^ with GA3 per day for 15, 30 or 45 days, respectively (n = 8) and groups 5, 6 and 7 (sham group) were received methanol alcohol (MA) without GA3 for 15, 30 or 45 days, respectively (n = 8).^14^

**Preparing GA3. **The required amount of the pure powder of GA3 (Merck, Darmstadt, Germany) has been weighed daily and then 100 mg of GA3 has been solved in 1 mL MA (Merck, Darmstadt, Germany) and normal saline has been added to reach its desired volume of 0.2 mL which was fed to each rat daily.

**Sperm collection. **At the end of each period, rats were euthanized with 75 mg kg^-1 ^ketamine (Alfasan, Woerden, The Netherlands) and 10 mg kg^-1 ^xylazine (Rotexmedica, Tritau, Germany), both IP. Through a caudal abdominal incision, the reproductive system was exposed. The left and right epididymis were carefully separated from the testis and placed in a petri dish containing 1 mL of HTF (Sigma, St. Louis, USA) medium with 4 mg mL^-1^ BSA (Sigma, St. Louis, USA). Cauda epididymis was minced to release sperms and then was placed in the incubator (37 ˚C) for 30 min, therefore, the sperms entered in the medium.^18^

**Epididymal sperm count. **Approximately 10 μL of the diluted sperm suspension (1:20) was transferred to each counting chamber of the hemocytometer and allowed to stand for 5 min. The settled cells during this time were counted by a light microscope at 200 × magnification. (The sperm heads were counted and expressed as million per mL of suspension).^19^

**Sperm viability assay. **Eosin-Nigrosin staining was used for this purpose. Firstly, a drop of medium containing sperm and a drop of Eosin were placed on a clean slide and mixed them together. After 30 sec, a drop of Nigrosin was added to it and smear was prepared and after drying at laboratory temperature, the percentage of live sperms was examined under a light microscope (Nikon, Tokyo, Japan). Meanwhile, 5 slides were prepared for each rat and 100 sperm per slide were counted under the light microscope.^19^

**Aniline blue staining. **At first, smear has been prepared from HTF medium sperms and after drying, fixed by the solution of 3% fixative glutaraldehyde for 30 min. Then, smears were stained by 5% aniline blue (using 4% acetic acid) for 5 to 8 min and the percentage of mature sperms (colorless head) and immature sperm (blue head) were observed by using a light microscope. Meanwhile, 5 slides were prepared for each rat and 100 sperm per slide were counted under the light.^20^

**Acridine orange staining (AO). **For assessment of sperm DNA integrity, air-dried smears were fixed overnight in methanol-glacial acetic acid (3:1) at room temperature. The slides were removed from the fixative and allowed to dry for a few min before staining with 0.19 mg mL^-1 ^and pH 2.5 AO (Sigma, St. Louis, USA) for 5 min at room temperature. Staining solution was prepared daily from a stock solution consisting of 1 mg AO in 1000 mL of distilled water and stored in the dark at 4 ˚C. To prepare the staining solution, 10 mL of the stock solution was added to 40 mL of 0.1 M citric acid (Sigma, St. Louis, USA) and 2.5 mL of 0.3 M Na_2_HPO_4_7H_2_O (Sigma, St. Louis, USA). All solutions were maintained at room temperature. After staining, the slides were gently rinsed in a stream of distilled water and sealed under a coverslip with nail polish. Meanwhile, 5 slides were prepared for each rat and 100 sperm per slide were counted under florescent microscope (Zeiss, Gottingen, Germany).^21^

**Oocyte collection. **Female rats were super ovulated with 15 IU of PMSG hormone and 15 IU of HCG hormone, both IP. After 10 to 12 hr of HCG injection, oocytes of rats were harvested through dissecting of oviduct.

The mR1ECM medium with 4 mg mL^-1 ^BSA was placed in the 5% CO_2 _incubator at 37 ˚C temperatures overnight. One fertilization drop (500 µL), two wash drops (150 µL) and two overnight culture drops(150 µL) of the medium were put into 3×3 cm petri dish and drops were covered by 9 mL mineral oil. The oocytes were put in the fertilization drop and then capacitated sperms (1 × 10^6^ per mL) were added to the fertilization drop. After 6 hr, the percentage of the fertilized oocytes was examined under invert microscope and fertilized oocytes (zygotes) were transferred to fresh medium after washing. Meanwhile the percentage of two-cell embryos and blastocysts were examined.^22^

**Preparation of mR1ECM medium: Stock A. **To prepare the stock, 0.239 g potassium chloride (Sigma, St. Louis, USA), 6.420 g sodium chloride (Sigma, St. Louis, USA), 1.352 g glucose (Sigma, St. Louis, USA), 0.075 g penicillin G (Sigma, St. Louis, USA), 0.050 g streptomycin (Sigma, St. Louis, USA), 1.900 mL sodium lactate (Sigma, St. Louis, USA) were dissolved in 100 mL distilled water and the solution was sterilized by filtration through 0.2 µm filter and stored at 4 ˚C as stock A.

**Preparation of mR1ECM medium: Stock B. **The reagents including 0.102 g magnesium chloride (Sigma, St. Louis, USA) and 0.294 g calcium chloride (Sigma, St. Louis, USA) were dissolved in distilled water and adjusted to the volume of 100 mL. The solution was sterilized by filtration through 0.200 µm filter and stored at 4 ˚C as stock B.

All reagents (10 mL Stock A, 10 mL Stock B, 0.210 g sodium bicarbonate (Sigma, St. Louis, USA), 0.0055 g sodium pyruvate (Sigma, St. Louis, USA), 0.0146 g L-glutamine (Sigma, St. Louis, USA), 2 mL of essential amino acids and 1 mL non-essential amino acids) were dissolved in distilled water and adjusted to the volume of 100 mL. The osmotic pressure was adjusted to about 310 mOsm. The solution was sterilized by filtration through 0.200 µm filter and stored at 4 ˚C.^23^

**Statistical analysis. **Statistical analysis was performed using one way analysis of variance (ANOVA). We used Bonferroni test, via the computer-based statistical programs SPSS (Version 19, IBM, Chicago, USA). A *p* value less than 0.05 was considered statistically significant.

## Results

**Sperm count. **According to the results of this study, it was determined that receiving GA3 could reduce the number of sperms (*p* < 0.05). It was found that total number of sperms in the GA3 groups gradually decreased over time and on days 30 and 45, there was a significant reduction compared with the control and MA groups (*p* < 0.05), (Table 1).

**Embryo development. **In the current research it was found that the groups in which the rats had received GA3, the fertility rate has decreased over the time. Mean of fertile oocytes in control, alcohol methanol 15, alcohol methanol 30, alcohol methanol 45, GA3 15, GA3 30 and GA3 45 groups were 67/92 (73%), 33/45 (74%), 63/84 (74%), 55/75 (73%), 43/64 (67%), 25/39 (64%) and 30/88, (34%), respectively. However, as seen in Table 1, it was determined that the fertility rate in GA3 groups on days 30 and 45 had significant (*p* < 0.05) differences with the control group, and also GA3 group on day 15. Mean of two-cell embryo in above mentioned groups were 57/67 (85%), 27/33 (81%), 49/63 (77%), 42/55 (76%), 31/43 (72%), 15/25 (58%) and 13/30 (43%) respectively. In this study, a small reduction, not statistically significant (*p* ≥ 0.05), in the percentage of two-cell embryos was seen in the MA groups compared to that of the control group on day 45, (Table 1).

**Table 1:** Average rate of fertilization parameters, percentage of two-cell embryos, blastocysts, sperm count, the percentage of immature sperm and percentage of sperm with chromatin damage in the control, GA3 and MA groups on days 15, 30 and 45 (Mean ± SE ).

**Groups**	**Sperms with damaged DNA (%)**	**Immature sperm (%)**	**Sperm viability ** **(%)**	**Sperm count (10** ^6^ **mL**^-1^**)**	**Blastocysts ** **(%)**	**Two-cell embryos (%)**	**Fertility rate (%)**
**Control**	9.00 ± 0.57^a^	9.00 ± 1.15^a^	88.66 ± 1.76^a^	48.33 ± 2.02^a^	72.33 ± 2.96^a^	85.00 ± 02.30^a^	73.00 ± 2.64^a^
**GA3 15**	11.00 ± 2.30^a^^b^	9.00 ± 1.52^a^	84.66 ± 4.05^a^	41.33 ± 2.40^a^	57.00 ± 4.93^a^	72.66 ± 2.73^a^	67.00 ± 1.76^a^
**GA3 30**	16.00 ± 1.52^b^	17.00 ± 0.57^b^	64.33 ± 4.33^b^	32.33 ± 2.40^b^	34.33 ± 3.37^b^	58.66 ± 3.27^b^	64.66 ± 4.47^a^
**GA3 45**	28.66 ± 2.40^c^	26.00 ± 01.15^c^	49.66 ± 1.44^c^	19.00 ± 1.15^c^	25.66 ± 2.90^b^	43.33 ± 3.52^b^	34.00 ± 3.05^b^
**MA 15**	8.66 ± 1.20^a^	8.00 ± 1.15^a^	87.00 ± 1.52^a^	48.33 ± 1.20^a^	66.66 ± 3.27^a^	81.00 ± 1.52^a^	74.00 ± 4.57^a^
**MA 30**	9.33 ± 1.44^a^	8.33 ± 0.87^a^	84.00 ± 1.73^a^	43.33 ± 0.87^a^	66.33 ± 2.40^a^	77.66 ± 2.18^a^	74.33 ± 3.27^a^
**MA 45**	8.66 ± 0.87^a^	8.00 ± 0.57^a^	82.66 ± 2.02^a^	45.00 ± 2.64^a^	67.33 ± 1.76^a^	76.33 ± 1.20^a^	73.33 ± 3.66^a^

abc Different letters indicate significant difference between groups in each column (*p* < 0.05).

In this research, according to Table 1, it is determined that the percentage of two-cell embryos in the GA3 groups at days 15, 30 and 45 shows significant (*p* < 0.05) differences with control and MA groups. It was also revealed that in MA groups there were no significant (*p* ≥ 0.05) differences in number of two-cell embryos with the control group.

**Fig. 1:**
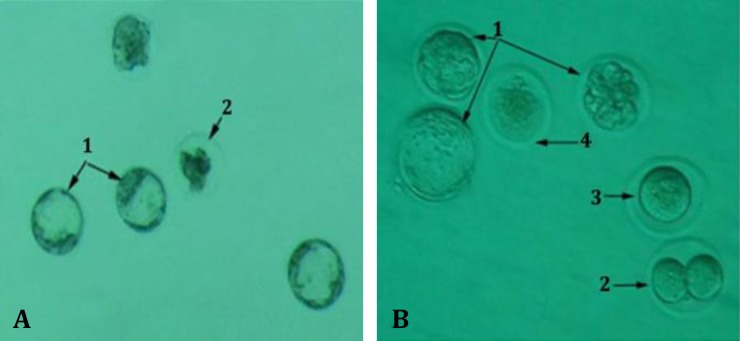
**A.** In the control group, several embryos were seen at the blastocyst stage (1) and a lysed embryo (2) on day 5 after incubation; **B. **In the GA3 group, three embryos at the blastocyst stage (1), one blocked embryo in two-cell stage (2), an unfertilized oocyte (3) and lysed oocyte (4) was observed (Invert microscope, 200×).

The mean value of blastocyst embryo in above mentioned groups were 41/57 (72%), 18/27 (66%), 32/49 (66%), 28/42 (67%), 18/31 (57%), 5/15 (34%) and 3/13 (25%), respectively. The percentage of blastocyst formation decreased in the groups of GA3 on days 30 and 45. Furthermore, a reduction in the percentage of blastocysts in MA groups on days 30 and 45 was seen, with no significant (*p* ≥ 0.05) difference with control group (Figs. 1A, 1B).

**Fig. 2:**
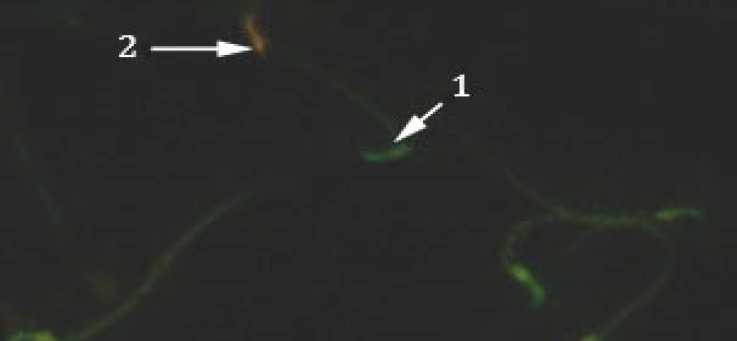
Sperm with healthy chromatin is seen green color (1) and sperms with orange color (2) head which have damaged chromatin in the GA3 group (AO, 400×).

**Evaluation of the sperm DNA integrity. **In AO staining, in order to investigate the sperms with abnormal DNA, we found that in the GA3 groups, the number of sperms with abnormal DNA has increased significantly on days 30 and 45 (*p* < 0.05) compared with the MA and control groups (Fig. 2).

**Fig. 3:**
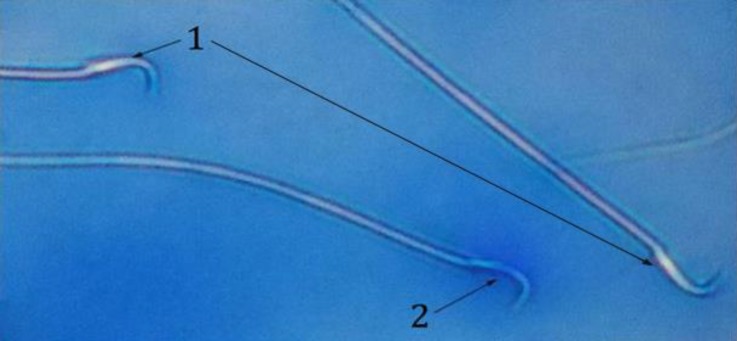
Two mature sperm (1) and one blue color immature sperm (2) in the GA3 group (Aniline blue, 400×).

**Immature sperm assay. **In aniline blue staining, it was found that the percentage of immature sperm in GA3 groups on days 30 and 45 has increased significantly (*p* < 0.05) compared with the MA and control groups (Fig. 3, Table 1). 

**Fig. 4:**
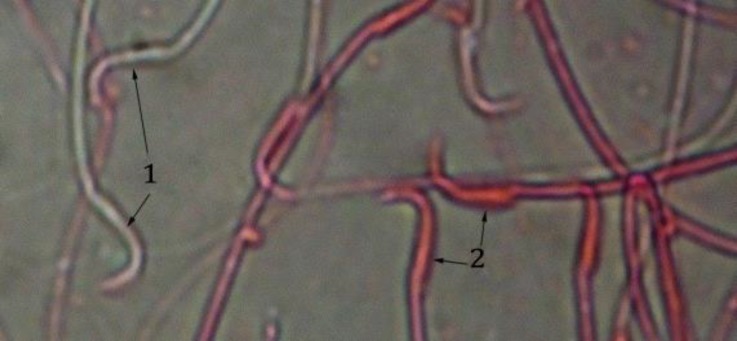
Live sperm (1) with discolored head, dead sperm (2) with a red head in the gibberellic acid group (Eosin and nigrosin, 400×).

**Sperm viability assay. **This study indicated that in the GA3 groups, the percentage of live sperms on days 30 and 45 decreased and showed a significant (*p* < 0.05) difference with MA and the control groups (Fig. 4).

## Discussion

In the present study GA3 caused a significant decrease in sperm count and the percentage of live sperm. The decrease in sperm count and percentage of live sperm in rats treated with GA3 were attributed to testosterone and ALP activity.^6^ The ALP is essential for metabolic processes providing energy for survival, motility and fertility of spermatozoa and has a role in synthesis of testosterone, and is necessary for the early stages in spermatogenesis.^24^^-^^26^ According to previous reports, the ALP has an important role in fertilization process.^27^ In male rabbits GA3 decreased significantly in serum testosterone concentration compared to control group.^28^ Alternatively, decreased sperm count may be due to decreased testosterone secretion as spermatogenesis process depends on the action of testosterone.^29^ The initiation of spermatogenesis at puberty and the maintenance of this process in adults are also depended on testosterone. Testosterone is required for the completion of meiosis and differentiation of the spermatids and maturation and conversion of round to elongated spermatids by promoting the stages VII and VIII of the spermatogenic cycle.^30^ It is confirmed that the conversion of round spermatids to their elongated forms was suppressed when testosterone in the interstitial fluid was only 5 % of the control group concentration.^31^

Since aniline blue staining reveals the existence of additional histone during histone-protamine replacement in testicular phase of sperm chromatin condensation therefore it can be said that the GA3 has affected this stage of the trend of spermatogenesis and immature sperms could increase. As mentioned, GA3 could decrease serum testosterone concentration^28^ and O’Donnell *et al*. proved that this hormone was required for sperm maturation.^30^

According to the results obtained from the current study, in AO staining, the number of sperms with denatured and abnormal DNA in GA3 group was more than control and alcohol groups whereas a minor and no significant increase was observed in methanol alcohol.

 In our study it was found that GA3 could increase immature sperms. We also found that the number of sperm with damaged chromatin increased significantly compared to that of the control and sham groups. Studies have shown that the percentage of sperms with abnormal chromatin and abnormal DNA is much more noticeable in infertile men than in fertile ones.^32^ Evenson *et al*. reported that if DNA-related aberrations were seen in more than 30% of sperms, that person could be considered as infertile.^33^

The increase in abnormal sperm with damaged chromatin could be due to oxidative stress. Pizarro *et al*. conclude that DNA damage caused by oxidative stress.^34^ The observations of Celik *et al*. led them to conclude that administration of subacute GA3 (50 and 100 ppm of PGRs as drinking water were administered orally to rats ad libitum for 25 days continuously) elevate malondialdehyde level and alter in the activities of antioxidative systems in rat’s various tissues.^13^ Our study also indicated that the malondialdehyde level increased in GA3 treatment group. Elevation in testis malondialdehyde levels due to GA3 may be due to that PGRs are associated with the generation of reactive oxygen species (ROS) which interacts with tissues leading to numerous pathophysiological alterations.^35^ The ROS could damage every major cellular component, including DNA.^36^^,^^37^

Our study on IVF showed that this hormone can reduce the percentage of fertilization rate, two-cell embryos and also the percentage of blastocysts by increasing immature sperms and sperms with damaged chromatin. A mechanism, by which GA3 reduced fertilization rate, might be explained through enhancement of ROS. It has been reported that ROS could modify the spermatozoon cytoskeleton and axoneme resulting in a reduction of sperm motility and the inhibition of sperm oocyte fusion, which in turn could lead to reduced fertility.^38^

In summary, we have demonstrated that GA3 could reduce the fertility in rats by influencing the sperm number, the number of live and immature sperms and DNA integrity of sperm’s chromatin.
